# Comprehensive registry of esophageal cancer in Japan, 2016

**DOI:** 10.1007/s10388-025-01141-8

**Published:** 2025-07-06

**Authors:** Yasue Kimura, Akihiko Okamura, Masashi Takeuchi, Hiroyuki Yamamoto, Hiroshi Saeki, Masayuki Watanabe, Ryu Ishihara, Masaki Ueno, Takashi Uno, Tsuneo Oyama, Koji Kono, Koji Tanaka, Takahiro Tsushima, Takeshi Toyozumi, Hodaka Numasaki, Hisahiro Matsubara, Tatsuya Miyazaki, Kei Muro, Masaru Morita, Shun Yamamoto, Toshiyuki Yoshio, Hiroya Takeuchi, The Registration Committee for Esophageal Cancer of the Japan Esophageal Society

**Affiliations:** 1 Department of Gastroenterological Surgery, National Hospital Organization Kyushu Cancer Center, 3-1-1 Notame, Minami-ku, Fukuoka, 811-1395 Japan; 2 Department of Gastroenterological Surgery, Cancer Institute Hospital of Japanese Foundation for Cancer Research, 3-8-31 Ariake, Koto-ku, Tokyo 135-8550 Japan; 3 Department of Surgery, Keio University School of Medicine, 35 Shinanomachi, Shinjyku-ku, Tokyo 160-8582 Japan; 4 Department of Healthcare Quality Assessment, Graduate School of Medicine, The University of Tokyo, 7-3-1 Hongo, Bunkyo-ku, Tokyo 113-8655 Japan; 5 Department of General Surgical Science, Graduate School of Medicine, Gunma University, 3-39-22 Showa-Machi, Maebashi, Gunma 371-8511 Japan; 6 Department of Gastrointestinal Oncology, Osaka International Cancer Institute, 3-1-69 Otemae, Chuo-ku, Osaka 541-8567 Japan; 7 Department of Gastroenterological Surgery, Toranomon Hospital, 2-2-2 Toranomon, Minato-ku, Tokyo 105-8470 Japan; 8 Department of Diagnostic Radiology and Radiation Oncology, Graduate School of Medicine, Chiba University, 1-8-1 Inohana, Chuo-ku, Chiba 260-8670 Japan; 9 Department of Endoscopy, Saku Central Hospital Advanced Care Center, 3400-28 Nakagomi, Saku, Nagano 385-0051 Japan; 10 Department of Gastrointestinal Tract Surgery, Fukushima Medical University School of Medicine, 1 Hikarigaoka, Fukushima, 960-1295 Japan; 11 Department Gastroenterological Surgery, Graduate School of Medicine, Osaka University, 2-2 Yamadaoka, Suita, Osaka 565-0871 Japan; 12 Division of Gastrointestinal Oncology, Shizuoka Cancer Center, 1007 Shimonagakubo, Nagaizumi-cho, Sunto-gun, Shizuoka, 411-8777 Japan; 13 Department of Frontier Surgery, Graduate School of Medicine, Chiba University, 1-8-1 Inohana, Chuo-ku, Chiba 260-8670 Japan; 14 Department of Medical Physics and Engineering, Graduate School of Medicine, Osaka University, 2-2 Yamadaoka, Suita, Osaka 565-0871 Japan; 15 Department of Surgery, Japanese Red Cross Maebashi Hospital, 389-1 Asakura-Machi, Maebashi, Gunma 371-0811 Japan; 16 Department of Clinical Oncology, Aichi Cancer Center Hospital, 1-1 Kanokoden, Chikusa-ku, Nagoya 464-8681 Japan; 17 Department of Head & Neck, Esophageal Medical Oncology, National Cancer Center Hospital, 5-1-1 Tsukiji, Cho-ku, Tokyo 104-0045 Japan; 18 Department of Upper Gastrointestinal Medicine, Cancer Institute Hospital of Japanese Foundation for Cancer Research, 3-8-31 Ariake, Koto-ku, Tokyo 135-8550 Japan; 19 Department of Surgery, Hamamatsu University School of Medicine, 1-20-1 Handayama, Chuo-ku, Hamamatsu, Shizuoka 431-3192 Japan

**Keywords:** Esophageal cancer, Comprehensive registry, Esophagectomy, Endoscopic treatment, Chemotherapy, Radiotherapy

## Abstract

**Background:**

The registration committee for esophageal cancer in the Japan Esophageal Society (JES) has collected the characteristics, treatments, and outcomes of patients who underwent any treatment in 2016 in Japan.

**Methods:**

We analyzed data on patients who had visited the participating hospitals in 2016. We collected the data using the National Clinical Database with a web-based data collection system. We used the Japanese Classification of Esophageal Cancer 11th edition by JES and the TNM Classification 8th edition by the Union of International Cancer Control (UICC) for cancer staging. Two committee members (A. O. and M. T.), endorsed by the Japan Esophageal Society and certified by the NCD, analyzed the data.

**Results:**

A total of 9,593 cases were registered from 347 institutions in Japan. Squamous cell carcinoma (SCC) and adenocarcinoma accounted for 87.5% and 7.2%, respectively. The 5-year survival rates of patients treated by endoscopic resection, concurrent chemoradiotherapy, radiotherapy alone, and esophagectomy were 89%, 36%, 26%, and 59%, respectively. Esophagectomy was performed in 4,988 cases. Minimally invasive approaches were selected for 65.1%, and 57.7% underwent thoracoscopic esophagectomy. The 5-year survival rates of esophagectomy cases with pStage 0, I, II, III, IVa, and IVb in the JES system were 83%, 79%, 67%, 40%, 34%, and 27%, respectively. In the UICC system, the survival of surgically resected SCC cases with pStage IVB, mainly due to supraclavicular lymph-node metastasis, was better than that with pStage IVA.

**Conclusion:**

We hope that this report improves all aspects of diagnosing and treating esophageal cancer in Japan.

**Supplementary Information:**

The online version contains supplementary material available at 10.1007/s10388-025-01141-8.

## Preface 2016

We sincerely appreciate the outstanding contributions of many physicians who participated in the esophageal cancer registry. The Comprehensive Registry of Esophageal Cancer in Japan, 2016, was published here. In 2019, the data collection method was changed from an electronic submission to a web-based data collection using the National Clinical Database (NCD). Personal information was replaced with individual management codes inside each institute, and the NCD collected only anonymized information. The data analysis had been performed in the NCD until the 2015 registry, while two committee members (A. O. and M. T.), endorsed by the Japan Esophageal Society and certified by the NCD, analyzed the data in this 2016 registry. This study was approved by the Ethics and Conflict of Interest Committee of National Clinical Database.

We briefly summarize the Comprehensive Registry of Esophageal Cancer in Japan 2016. According to the subject year, we used the Japanese Classification of Esophageal Cancer 11th by the Japan Esophageal Society (JES) [1, 2] and the Union of International Cancer Control (UICC) TNM Classification 8th [3] for cancer staging. A total of 9,593 cases were registered from 347 institutions in Japan. Tumor locations were cervical in 5.2%, upper thoracic in 12.5%, middle thoracic in 46.4%, lower thoracic in 26.7%, and esophagogastric junction in 8.1%. Superficial carcinomas (Tis, T1a, and T1b) were 40.0%. As for the histologic type of biopsy specimens, squamous cell carcinoma (SCC) and adenocarcinoma (ADC) accounted for 87.5% and 7.2%, respectively. Regarding clinical results, the 5-year survival rates of patients treated using endoscopic resection, concurrent chemoradiotherapy, radiotherapy alone, and esophagectomy were 89%, 36%, 26%, and 59%, respectively. The endoscopic submucosal dissection accounted for 92.1% of curative-intent endoscopic treatment. Esophagectomy was performed in 4,988 cases. Minimally invasive approaches were selected for 65.1% and 57.7% underwent thoracoscopic esophagectomy.

In the JES classification, the survival curves declined according to the progress in the pStage after esophagectomy in order. In the UICC classification, different staging systems were adopted for SCC and ADC. In the surgically resected SCC cases, the survival of pStage IVB patients was better than that of pStage IVA, as many pStage IVB patients were classified as this stage due to the supraclavicular lymph-node metastasis. In the surgically resected ADC cases, the survival curves were almost identical between pStage IIIA and IIIB and pStage IVA and IVB.

We hope that this Comprehensive Registry of Esophageal Cancer in Japan 2016 will help improve all aspects of diagnosing and treating esophageal cancer in Japan.

I. Clinical factors of esophageal cancer patients treated in 2016.
  1. Institutions participated in the 2016 registry
  2. Overall results

Table 1. Patient characteristics.

**Table 1:** Patient characteristics

		*n*	%
Sex	Female	1,647	17.2
	Male	7,946	82.8
Age	≤ 29	6	0.1
	30–39	24	0.3
	40–49	272	2.8
	50–59	1,221	12.7
	60–69	3,596	37.5
	70–79	3,468	36.2
	80–89	960	10.0
	90 ≤	37	0.4
	Unknown	9	0.1
Tumor location	Cervical	500	5.2
	Upper thoracic	1,199	12.5
	Middle thoracic	4,450	46.4
	Lower thoracic	2,560	26.7
	EG	556	5.8
	E = G	129	1.3
	GE	100	1.0
	Unknown	99	1.0
Macroscopic tumor type	Type 0-Ip	52	0.5
	Type 0-Is	228	2.4
	Type 0-IIa	776	8.1
	Type 0-IIb	876	9.1
	Type 0-IIc	2,104	21.9
	Type 0-III	34	0.4
	Type 1	718	7.5
	Type 2	2,474	25.8
	Type 3	1,610	16.8
	Type 4	91	1.0
	Type 5a	101	1.1
	Type 5b	63	0.7
	Unknown	466	4.9
Histologic types of biopsy specimens	Squamous cell carcinoma	8,396	87.5
	Adenocarcinoma	692	7.2
	(Barrett's carcinoma)	171	1.8
	Basaloid carcinoma	31	0.3
	Neuroendocrine carcinoma	58	0.6
	Neuroendocrine tumor	2	0.0
	Adenosquamous carcinoma	19	0.2
	Malignant melanoma	24	0.3
	Carcinosarcoma	22	0.2
	Undifferentiated carcinoma	8	0.1
	Mucoepidermoid carcinoma	3	0.0
	Adenoid cystic carcinoma	1	0.0
	Other epithelial tumors	36	0.4
	GIST	7	0.1
	Nonepithelial tumors	4	0.0
	Other tumors	61	0.6
	Unknown	229	2.4
Depth of tumor invasion (UICC 8th)	cT0	18	0.2
	cTis	343	3.6
	cT1a	1,664	17.4
	cT1b	1,833	19.1
	cT2	1,090	11.4
	cT3	3,296	34.4
	cT4a	289	3.0
	cT4b	884	9.2
	cTX	176	1.8
Lymph-node metastasis (UICC 8th)	cN0	4,909	51.2
	cN1	2,609	27.2
	cN2	1,616	16.9
	cN3	458	4.8
	cNX	1	0.0
Distant metastasis (UICC 8th)	cM0	8,677	90.5
	cM1	916	9.6
Clinical stage (UICC 8th)	cStage 0	306	3.2
	cStage I	3,254	33.9
	cStage II	1,383	14.4
	cStage IIA	13	0.1
	cStage IIB	68	0.7
	cStage III	2,182	22.8
	cStage IVA	1,028	10.7
	cStage IVB	833	8.7
	Unknown	141	1.5
Performed treatment	Surgery	5,075	55.7
	Chemotherapy	4,702	51.7
	Radiotherapy	2,538	27.9
	Endoscopic treatment	2,026	22.3
	Others	133	1.5
Multi-organ primary cancer*	None	6,762	70.5
	Synchronous cancer	1,260	13.1
	Prior to esophageal cancer	1,277	13.3
	Subsequent to esophageal cancer	609	6.4
	Unknown	34	0.4

* Details are shown in Supplemental Table 1

Table 1. Multiple primary cancer sites.

**Fig. 1:**
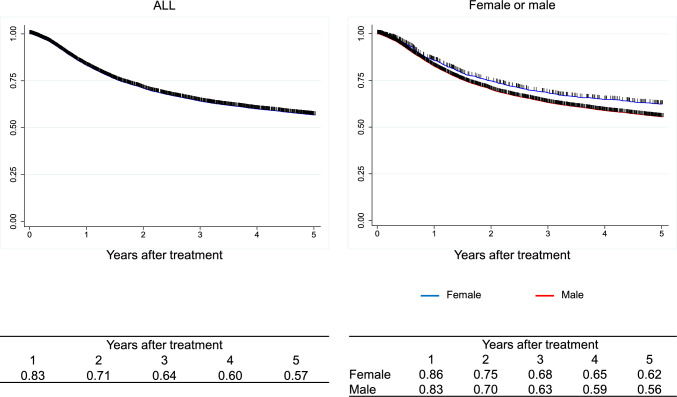
Survival of all registered patients

Figure 1. Survival of all registered patients.

**Fig. 2:**
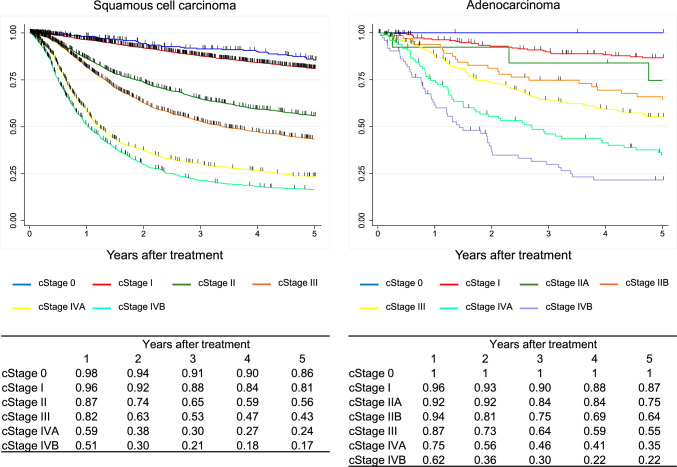
Survival of all registered patients according to the clinical stage (UICC 8.^th^)

Figure 2. Survival of all registered patients according to the clinical stage (UICC 8^th^)

- II. Results of endoscopically treated patients in 2016.

Table 2. Details of endoscopic treatment.

**Table 2:** Details of endoscopic treatment

		*n*	%
Curative intent		1,945	
	EMR	126	6.5
	ESD	1,792	92.1
	PDT	9	0.5
	YAG laser	36	1.9
	MCT/RFA	1	0.1
	Others	23	1.2
	Unknown	2	0.1
Palliative intent		256	
	YAG laser	8	3.1
	Esophageal stent	126	49.2
	Tracheobronchial stent	5	2.0
	Unknown	117	45.7
EMR endoscopic mucosal resection, ESD endoscopic submucosal dissection, PDT photodynamic therapy, YAG yttrium aluminum garnet, MCT microwave coagulation therapy, RFA radiofrequency ablation			

Table 3. Characteristics of patients treated with EMR/ESD.

**Table 3:** Characteristics of patients treated with EMR/ESD

		*n*	%
Sex	Female	305	16.0
	Male	1,604	84.0
Age	≤ 29	1	0.1
	30–39	3	0.2
	40–49	42	2.2
	50–59	231	12.1
	60–69	702	36.8
	70–79	712	37.3
	80–89	214	11.2
	90 ≤	2	0.1
	Unknown	2	0.1
Tumor location	Cervical	49	2.6
	Upper thoracic	222	11.6
	Middle thoracic	1,025	53.7
	Lower thoracic	460	24.1
	EG	97	5.1
	E = G	20	1.1
	GE	7	0.4
	Unknown	29	1.5
Macroscopic tumor type	Type 0-Ip	8	0.4
	Type 0-Is	32	1.7
	Type 0-IIa	244	12.8
	Type 0-IIb	514	26.9
	Type 0-IIc	1,034	54.2
	Type 0-III	5	0.3
	Type 1	5	0.3
	Type 2	14	0.7
	Type 3	7	0.4
	Type 4	0	0.0
	Type 5a	3	0.2
	Type 5b	2	0.1
	Unknown	41	2.2
Histologic types of biopsy specimens	Squamous cell carcinoma	1,636	85.7
	Adenocarcinoma	110	5.8
	(Barrett's carcinoma)	53	2.8
	Basaloid carcinoma	1	0.1
	Neuroendocrine tumor	1	0.1
	Adenosquamous carcinoma	2	0.1
	Mucoepidermoid carcinoma	1	0.1
	Other epithelial tumors	19	1.0
	Nonepithelial tumors	1	0.1
	Other tumors	42	2.2
	Unknown	96	5.0
Depth of tumor invasion (UICC 8th)	cT0	6	0.3
	cTis	300	15.7
	cT1a	1,298	68.0
	cT1b	250	13.1
	cT2	6	0.3
	cT3	18	0.9
	cT4a	2	0.1
	cT4b	3	0.2
	cTX	26	1.4
Lymph-node metastasis (UICC 8th)	cN0	1,875	98.2
	cN1	22	1.2
	cN2	10	0.5
	cN3	2	0.1
Distant metastasis (UICC 8th)	cM0	1,906	99.8
	cM1	3	0.2
Clinical stage (UICC 8th)	cStage 0	258	13.5
	cStage I	1,465	76.7
	cStage II	7	0.4
	cStage IIA	1	0.1
	cStage IIB	1	0.1
	cStage III	14	0.7
	cStage IVA	4	0.2
	cStage IVB	3	0.2
	Unknown	16	0.8

Table 4. Detailed of EMR/ESD.

**Table 4:** Details of EMR/ESD

		*n*	%
Type of treatment	EMR	118	6.2
	ESD	1,791	93.8
Curativity	One-piece complete resection	1,821	95.4
	One-piece incomplete resection (HM1)	29	1.5
	One-piece incomplete resection (VM1)	15	0.8
	Divided resection	38	2.0
	None (interruption)	1	0.1
	Unknown	5	0.3
Complications	None	1,831	95.9
	Perforation	20	1.1
	Bleeding	5	0.3
	Mediastinitis	7	0.4
	Stenosis	44	2.3
	Others	12	0.6
	Unknown	3	0.2

Table 5. Pathologic findings of EMR/ESD specimens.

**Table 5:** Pathologic findings of EMR/ESD specimens

		*n*	%
Histologic types	Squamous cell carcinoma	1,745	91.4
	Adenocarcinoma	111	5.8
	(Barrett's carcinoma)	53	2.8
	Basaloid carcinoma	4	0.2
	Neuroendocrine carcinoma	1	0.1
	Adenosquamous carcinoma	4	0.2
	Mucoepidermoid carcinoma	2	0.1
	Other epithelial tumors	16	0.8
	Nonepithelial tumors	1	0.1
	Other tumors	17	0.9
	Unknown	8	0.4
Depth of tumor invasion (UICC 8th)	pT1a-EP	493	25.8
	pT1a-LPM	740	38.8
	pT1a-MM	293	15.4
	pT1b-SM1	107	5.6
	pT1b-SM2	197	10.3
	pT1b-SM3	4	0.2
	pT2 or more	24	1.3
	pTX	51	2.7
Lymphatic invasion	Positive	174	9.1
	Negative	1,689	88.5
	Unknown	46	2.4
Venous invasion	Positive	113	5.9
	Negative	1,750	91.7
	Unknown	46	2.4
Horizontal margin	HM1	100	5.2
	HM0	1,670	87.5
	Unknown	139	7.3
Vertical margin	VM1	39	2.0
	VM0	1,816	95.1
	Unknown	54	2.8

**Fig. 3:**
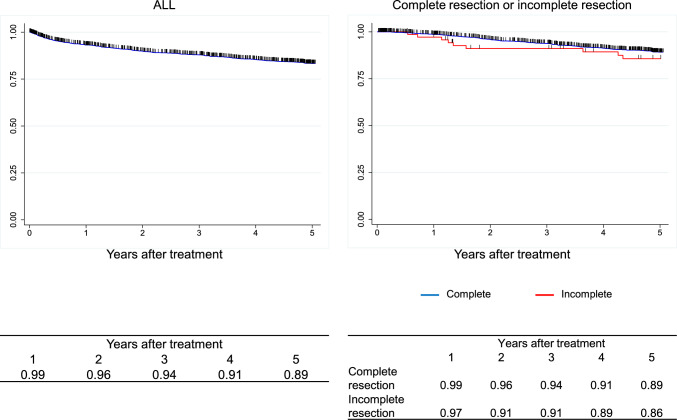
Overall survival of patients treated with EMR/ESD

Figure 3 Overall survival of patients treated with EMR/ESD.

**Fig. 4:**
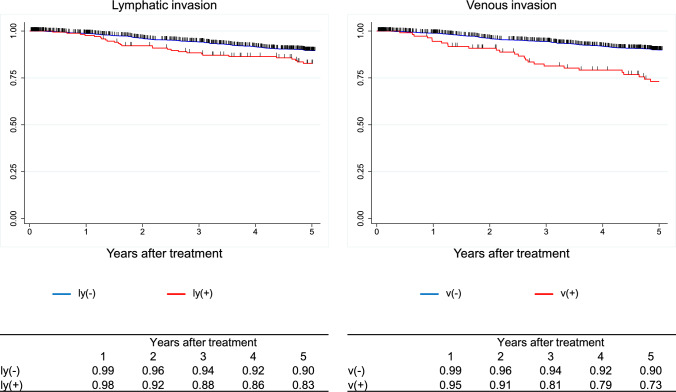
Overall survival of patients treated with EMR/ESD according to lymphovascular invasion

Figure 4. Overall survival of patients treated with EMR/ESD according to lymphovascular invasion

III. Results in patients treated with chemotherapy in 2016.

Table 6. Details of chemotherapy

**Table 6:** Details of chemotherapy

		*n*	%
Objective	Unresectable	1,791	35.2
	Neoadjuvant	2,476	48.6
	Intraoperative	2	0.0
	Adjuvant	661	13.0
	Residual disease	92	1.8
	Recurrence	553	10.9
	Others	0	0.0
Treatment type	Chemotherapy alone	3,025	59.4
	Chemoradiotherapy(concurrent)	1,816	35.7
	Chemoradiotherapy(sequential)	230	4.5
	Others	20	0.4
	Unknown	3	0.1

Table 7. Characteristics of patients treated with chemotherapy for unresectable diseases

**Table 7:** Characteristics of patients with chemotherapy for unresectable diseases

		*n*	%
Sex	Female	284	15.9
	Male	1,507	84.1
Age	≤ 29	1	0.1
	30–39	4	0.2
	40–49	56	3.1
	50–59	207	11.6
	60–69	660	36.9
	70–79	676	37.7
	80–89	183	10.2
	90 ≤	2	0.1
	Unknown	2	0.1
Tumor location	Cervical	214	12.0
	Upper thoracic	287	16.0
	Middle thoracic	811	45.3
	Lower thoracic	383	21.4
	EG	62	3.5
	E = G	12	0.7
	GE	9	0.5
	Unknown	13	0.7
Macroscopic tumor type	Type 0-Ip	3	0.2
	Type 0-Is	28	1.6
	Type 0-IIa	78	4.4
	Type 0-IIb	61	3.4
	Type 0-IIc	176	9.8
	Type 0-III	2	0.1
	Type 1	175	9.8
	Type 2	672	37.5
	Type 3	449	25.1
	Type 4	20	1.1
	Type 5a	18	1.0
	Type 5b	1	0.1
	Unknown	108	6.0
Histologic types of biopsy specimens	Squamous cell carcinoma	1,641	91.6
	Adenocarcinoma	68	3.8
	Neuroendocrine tumor	1	0.1
	Adenosquamous carcinoma	1	0.1
	Malignant melanoma	5	0.3
	Carcinosarcoma	2	0.1
	Undifferentiated carcinoma	3	0.2
	Other epithelial tumors	7	0.4
	Other tumors	5	0.3
	Unknown	28	1.6
Depth of tumor invasion (UICC 8th)	cT0	0	0.0
	cTis	3	0.2
	cT1a	46	2.6
	cT1b	235	13.1
	cT2	158	8.8
	cT3	675	37.7
	cT4a	117	6.5
	cT4b	535	29.9
	cTX	22	1.2
Lymph-node metastasis (UICC 8th)	cN0	407	22.7
	cN1	598	33.4
	cN2	547	30.5
	cN3	239	13.3
Distant metastasis (UICC 8th)	cM0	1,228	68.6
	cM1	563	31.4
Clinical stage (UICC 8th)	cStage 0	1	0.1
	cStage I	243	13.6
	cStage II	159	8.9
	cStage III	305	17.0
	cStage IVA	467	26.1
	cStage IVB	535	29.9
	Unknown	18	1.0
Objective	Curative intent	1,434	80.1
	Palliative intent	357	19.9

**Fig. 5:**
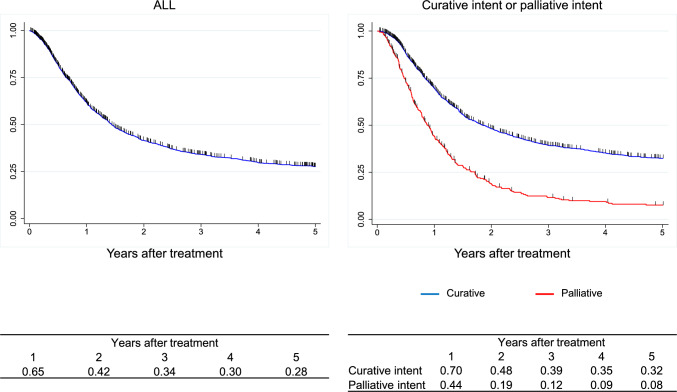
Overall survival of patients treated with chemotherapy, including chemoradiotherapy for unresectable diseases

Figure 5. Overall survival of patients treated with chemotherapy, including chemoradiotherapy for unresectable diseases

IV. Results in patients treated with radiotherapy in 2016.

Table 8. Details of radiotherapy

**Table 8:** Details of radiotherapy

		*n*	%
Objective	Recurrence	399	14.1
	Definitive	1,474	51.9
	Palliative	413	14.6
	Preoperative	324	11.4
	Postoperative	127	4.5
	Residual	83	2.9
	Others	17	0.6
	Unknown	1	0.0
Concurrent chemotherapy	Done	2,236	78.8
	None	597	21.0
	Unknown	5	0.2
External irradiation	Done	2,702	95.2
	Not done	136	4.8
Intracavitary irradiation	Done	2	0.1
	Not done	2,799	98.6
	Unknown	37	1.3
Target volume	Primary tumor	2,272	80.1
	Neck + upper mediastinum	970	34.2
	Supraclavicular nodes	794	28.0
	Mediastinal nodes	1,447	51.0
	Abdominal nodes	679	23.9
	Distant metastasis	144	5.1
Prophylactic irradiation for regional nodes	Done	993	35.0
	Not done	1,687	59.4
	Unknown	158	5.6
Irradiation device	High-energy X-ray	2,725	96.0
	Proton beam	29	1.0
	Cobalt	4	0.1
	Heavy particle beam	2	0.1
	Unknown	78	2.8
Irradiation method	3D	2,162	76.2
	IMRT	401	14.1
	2D	197	6.9
Dose of external irradiation	≤ 29	115	4.3
	30–39	139	5.1
	40–49	437	16.2
	50–59	525	19.4
	60–69	1,425	52.7
	70 ≤	46	1.7
	Unknown	15	0.6
Number of fraction	≤ 29	1,189	44.0
	30–39	1,448	53.6
	40–49	23	0.9
	50–59	7	0.3
	60 ≤	3	0.1
	Unknown	32	1.2

**Fig. 6:**
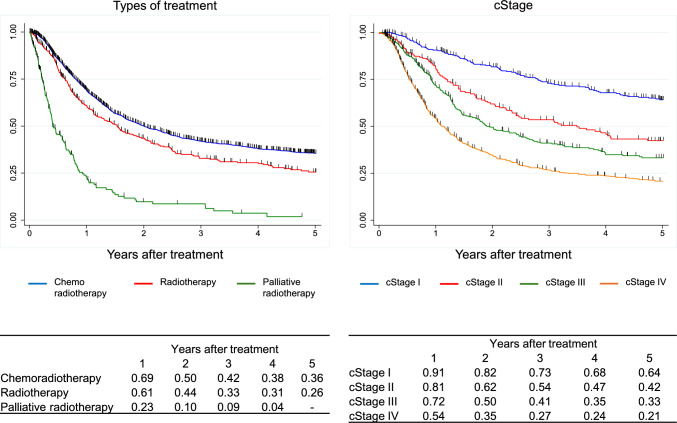
Overall survival of patients treated with radiotherapy

Figure 6. Overall survival of patients treated with radiotherapy

V. Results in patients who underwent esophagectomy in 2016.

Table 9. Types of surgery

**Table 9:** Types of surgery

	*n*	%
Esophagectomy with one-stage reconstruction	4,829	92.1
Esophagectomy with staged reconstruction	125	2.4
Esophagectomy without reconstruction	34	0.6
Bypass	71	1.4
Exploratory thoracotomy/laparotomy	22	0.4
Stoma	47	0.9
Esophagostomy	1	0.0
Lymphadenectomy	26	0.5
Others	90	1.7

Table 10. Characteristics of patients who underwent esophagectomy

**Table 10:** Characteristics of patients who underwent esophagectomy

		*n*	%
Sex	Female	901	18.1
	Male	4,087	81.9
Age	≤ 29	3	0.1
	30–39	14	0.3
	40–49	164	3.3
	50–59	727	14.6
	60–69	2,026	40.7
	70–79	1,790	35.9
	80–89	254	5.1
	90 ≤	5	0.1
	Unknown	5	0.1
Tumor location	Cervical	170	3.4
	Upper thoracic	581	11.7
	Middle thoracic	2,191	43.9
	Lower thoracic	1,507	30.2
	EG	363	7.3
	E = G	85	1.7
	GE	73	1.5
	Unknown	13	0.7
Macroscopic tumor type	Type 0-Ip	42	0.8
	Type 0-Is	157	3.2
	Type 0-IIa	409	8.2
	Type 0-IIb	250	5.0
	Type 0-IIc	857	17.2
	Type 0-III	23	0.5
	Type 1	451	9.0
	Type 2	1,494	30.0
	Type 3	997	20.0
	Type 4	50	1.0
	Type 5a	74	1.5
	Type 5b	58	1.2
	Unknown	126	2.5
Histologic types of biopsy specimens	Squamous cell carcinoma	4,348	87.2
	Adenocarcinoma	470	9.4
	(Barrett's carcinoma)	108	2.2
	Basaloid carcinoma	24	0.5
	Neuroendocrine carcinoma	26	0.5
	Neuroendocrine tumor	1	0.0
	Adenosquamous carcinoma	15	0.3
	Malignant melanoma	17	0.3
	Carcinosarcoma	20	0.4
	Undifferentiated carcinoma	3	0.1
	Mucoepidermoid carcinoma	2	0.0
	Adenoid cystic carcinoma	1	0.0
	Other epithelial tumors	10	0.2
	GIST	4	0.1
	Nonepithelial tumors	4	0.1
	Other tumors	8	0.2
	Unknown	35	0.7
Depth of tumor invasion (UICC 8th)	cT0	9	0.2
	cTis	9	0.2
	cT1a	270	5.4
	cT1b	1,301	26.1
	cT2	818	16.4
	cT3	2,262	45.4
	cT4a	121	2.4
	cT4b	181	3.6
	cTX	17	0.3
Lymph-node metastasis (UICC 8th)	cN0	2,290	45.9
	cN1	1,703	34.1
	cN2	864	17.3
	cN3	130	2.6
	cNX	1	0.0
Distant metastasis (UICC 8th)	cM0	4,812	96.5
	cM1	176	3.5
Clinical sage (UICC 8th)	cStage 0	15	0.3
	cStage I	1,475	29.6
	cStage II	1,067	21.4
	cStage IIA	8	0.2
	cStage IIB	64	1.3
	cStage III	1,689	33.9
	cStage IVA	387	7.8
	cStage IVB	174	3.5
	Unknown	14	0.3
Treatment type	Upfront surgery	1,823	36.6
	Preoperative chemotherapy + surgery	1,962	39.3
	Preoperative chemoradiotherapy + surgery	324	6.5
	Surgery + postoperative chemotherapy	503	10.1
	Others	376	7.5

Table 11. Approach in esophagectomy

**Table 11:** Approach in esophagectomy

		*n*	%
Approach	Right thoracic	4,221	84.6
	Left thoracoabdominal	74	1.5
	Left thoracic	54	1.1
	Transhiatal thoracic esophagectomy	61	1.2
	Transhiatal lower esophagectomy	95	1.9
	Sternotomy	2	0.0
	Abdominal	132	2.7
	Cervical	306	6.1
	Others	29	0.6
	Unknown	14	0.3
Video-assisted surgery	Video-assisted surgery	3,247	65.1
	Thoracoscopy	2,877	88.6
	Laparoscopy	1,614	49.7
	Mediastinoscopy	141	4.3
	Others	25	0.8
	None	1,736	34.8
	Unknown	5	0.1

Table 12. Extent and degree of lymph-node dissection in esophagectomy

**Table 12:** Extent and degree of lymph-node dissection in esophagectomy

		*n*	%
Extent	None	89	1.8
	Done	4,899	98.2
	Bilateral cervical nodes	2,620	53.5
	Upper mediastinal nodes	4,267	87.1
	Middle-lower mediastinal nodes	4,599	93.9
	Abdominal nodes	4,572	93.3
Degree	D0	189	4
	D1	504	10
	D2	2,404	48
	D3	1,823	37
	DX	68	1

Table 13. Details of reconstruction

**Table 13:** Details of reconstruction

		*n*	%
Reconstruction route	Retrosternal	2,334	46.7
	Posterior mediastinal	2,046	40.9
	Subcutaneous	304	6.1
	Intrathoracic	185	3.7
	Cervical	46	0.9
	Others	28	0.6
	None	48	1.0
	Unknown	8	0.2
Sites of anastomosis	Neck	4,073	81.5
	Proximal thoracic cavity	461	9.2
	Distal thoracic cavity	237	4.7
	Lower mediastinum	101	2.0
	Subcutaneous	38	0.8
	Others	18	0.4
	None	46	0.9
	Unknown	25	0.5
Organs for substitution	Whole stomach	207	4.2
	Gastric tube	4,263	86.5
	Pedicled jejunum	229	4.7
	Free jejunum	106	2.2
	Pedicled colon	125	2.5
	Free colon	9	0.2
	Skin roll	1	0.0
	Others	38	0.8
	None	71	1.4
Vascular anastomosis for reconstruction	Not done	2,361	90.6
	Done	148	5.7
	Unknown	98	3.8

Table 14. Invaded organs and combined resected organs in esophagectomy

**Table 14:** Invaded organs and combined resected organs in esophagectomy

		*n*	%
Invaded organs	Trachea	81	22.6
	Bronchi	46	12.8
	Lung	36	10.0
	Aorta	44	12.3
	Vena cava	13	3.6
	Pericardium	35	9.8
	Diaphragm	52	14.5
	Stomach	29	8.1
	Liver	22	6.1
	Pancreas	11	3.1
	Thyroid gland	11	3.1
	Others	62	17.3
Combined resected organs	Pharynx	56	5.5
	Larynx	71	7.0
	Trachea	37	3.7
	Aorta	26	2.6
	Lung	61	6.0
	Pericardium	28	2.8
	Diaphragm	47	4.6
	Stomach	226	22.3
	Pancreas + spleen	12	1.2
	Recurrent nerve	52	5.1
	Vagal nerve (main trunk)	5	0.5
	Others	243	24.0

Table 15. Pathologic findings of surgically resected specimens

**Table 15:** Pathologic findings of surgically resected specimens

		*n*	%
Histologic types	Squamous cell carcinoma	4,172	83.6
	Adenocarcinoma	460	9.2
	(Barrett's carcinoma)	113	2.3
	Basaloid carcinoma	63	1.3
	Neuroendocrine carcinoma	44	0.9
	Neuroendocrine tumor	1	0.0
	Adenosquamous carcinoma	23	0.5
	Malignant melanoma	17	0.3
	Carcinosarcoma	36	0.7
	Undifferentiated carcinoma	3	0.1
	Mucoepidermoid carcinoma	1	0.0
	Adenoid cystic carcinoma	2	0.0
	Other epithelial tumors	3	0.1
	GIST	4	0.1
	Nonepithelial tumors	5	0.1
	Other tumors	38	0.8
	Unknown	116	2.3
Depth of tumor invasion (UICC 8th)	pT0	200	4.0
	pTis	22	0.4
	pT1a	621	12.5
	pT1b	1,384	27.8
	pT2	626	12.6
	pT3	1,839	36.9
	pT4a	141	2.8
	pT4b	108	2.2
	pTX	47	0.9
Lymph-node metastasis (UICC 8th)	pN0	2,479	49.7
	pN1	1,342	26.9
	pN2	746	15.0
	pN3	370	7.4
	pNX	51	1.0
Distant metastasis (UICC 8th)	cM0	4,596	92.1
	cM1	18	0.4
	pM1	374	7.5
Pathological stage (UICC 8th)	pStage 0	84	1.7
	pStage IA	507	10.2
	pStage IB	831	16.7
	pStage IIA	273	5.5
	pStage IIB	932	18.7
	pStage IIIA	273	5.5
	pStage IIIB	1,045	21.0
	pStage IVA	352	7.1
	pStage IVB	371	7.4
	Unknown	95	1.9
Depth of tumor invasion (JES 11th)	pT0	199	4.0
	pT1a	634	12.7
	pT1b	1,389	27.9
	pT2	630	12.6
	pT3	1,840	36.9
	pT4a	140	2.8
	pT4b	99	2.0
	Unknown	57	1.1
Lymph-node metastasis (JES 11th)	N0	2,441	48.9
	N1	892	17.9
	N2	976	19.6
	N3	381	7.6
	N4	271	5.4
	Unknown	27	0.5
Distant metastasis (JES 11th)	cM0	4,851	97.3
	pM1	90	1.8
	Unknown	47	0.9
Pathological stage (JES 11th)	pStage 0	681	13.7
	pStage I	846	17.0
	pStage II	1,460	29.3
	pStage III	1,469	29.5
	pStage IVa	321	6.4
	pStage IVb	88	1.8
	Unknown	123	2.5
Lymphatic invasion	ly1-3	2,221	44.5
	ly0	2,623	52.6
	Unknown	144	2.9
Venous invasion	v1-3	2,223	44.6
	v0	2,620	52.5
	Unknown	145	2.9
Infiltrative growth pattern	INFa	647	13.0
	INFb	3,216	64.5
	INFc	260	5.2
	Unknown	865	17.3
Intramural metastasis	pIM1	186	3.7
	pIM0	4,576	91.7
	Unknown	226	4.5
Intramural metastasis in the gastric wall	pIM1-St	49	1.0
	pIM0-St	4,679	93.8
	Unknown	260	5.2
Histological response of chemotherapy and/or radiotherapy	Grade0	190	3.8
	Grade1a	1,280	25.7
	Grade1b	388	7.8
	Grade2	445	8.9
	Grade3	222	4.5
	No preoperataive treatment	2,052	41.1
	Unknown	411	8.2
Residual tumor	R0	4,504	90.3
	R1	238	4.8
	R2	138	2.8
	RX	108	2.2
Proximal and distal margin	pPM1 or pDM1	106	2.1
	pPM0 and pDM0	4,796	96.2
	Unknown	86	1.7
Radial margin	pRM1	268	5.4
	pRM0	4,483	89.9
	Unknown	237	4.8
Curativity	pCurA	4,212	84.4
	pCurB	593	11.9
	pCurC	183	3.7

**Fig. 7:**
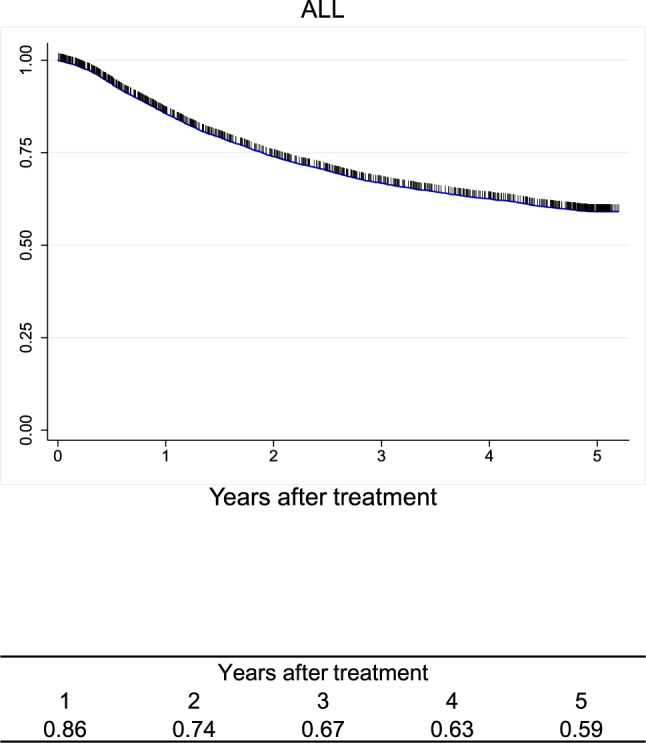
Overall survival of patients who underwent esophagectomy

Figure 7. Overall survival of patients who underwent esophagectomy

**Fig. 8:**
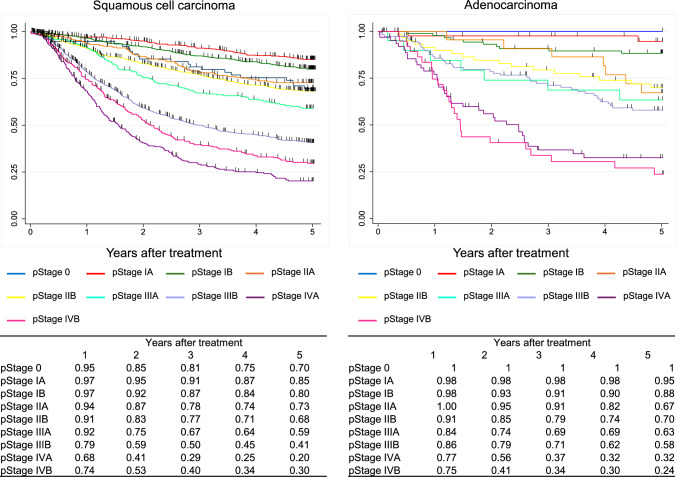
Overall survival of patients who underwent esophagectomy according to pathological stage (UICC 8.^th^)

Figure 8. Overall survival of patients who underwent esophagectomy according to pathological stage (UICC 8^th^)

**Fig. 9:**
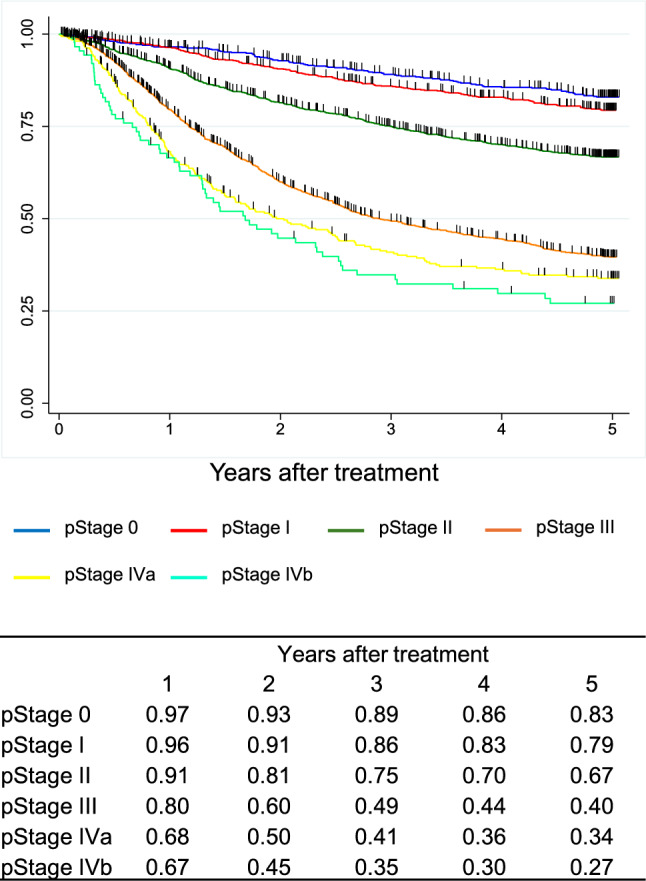
Overall survival of patients who underwent esophagectomy according to pathological stage (JES 11.^th^)

Figure 9. Overall survival of patients who underwent esophagectomy according to pathological stage (JES 11^th^)

**Fig. 10:**
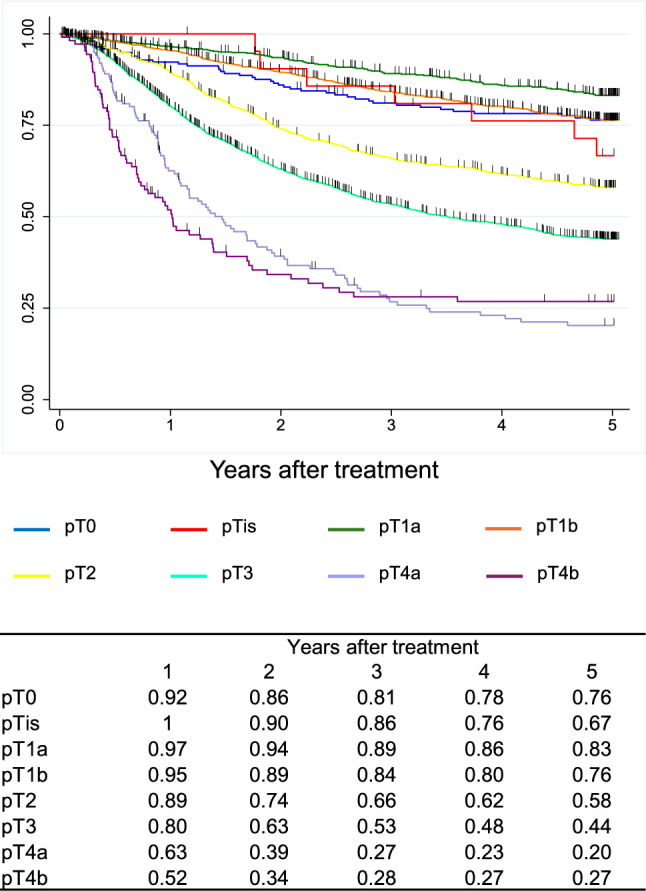
Overall survival of patients who underwent esophagectomy according to depth of tumor invasion (UICC 8.^th^)

Figure 10. Overall survival of patients who underwent esophagectomy according to depth of tumor invasion (UICC 8^th^)

**Fig. 11:**
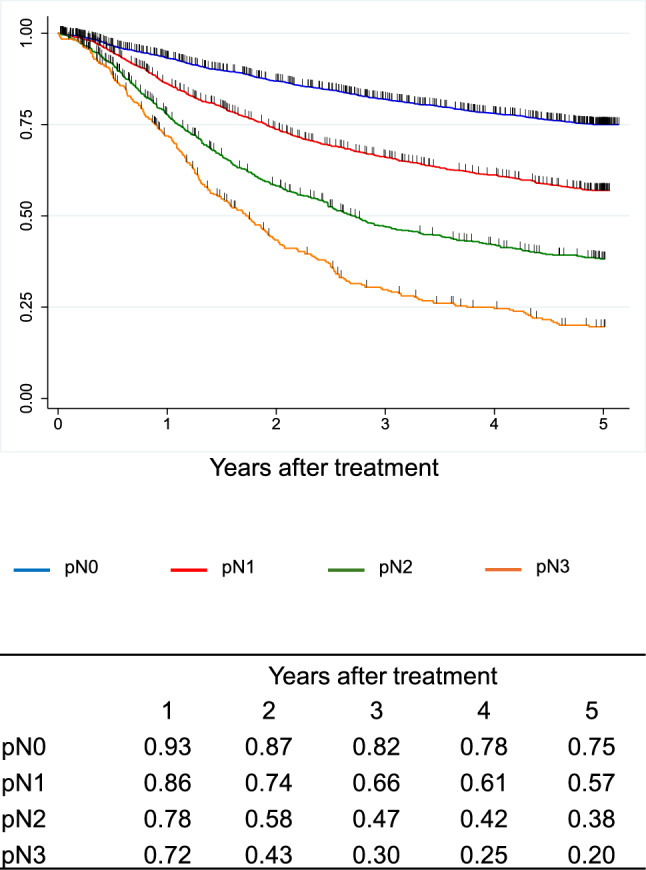
Overall survival of patients who underwent esophagectomy according to lymph-node metastasis (UICC 8.^th^)

Figure 11. Overall survival of patients who underwent esophagectomy according to lymph-node metastasis (UICC 8^th^)

**Fig. 12:**
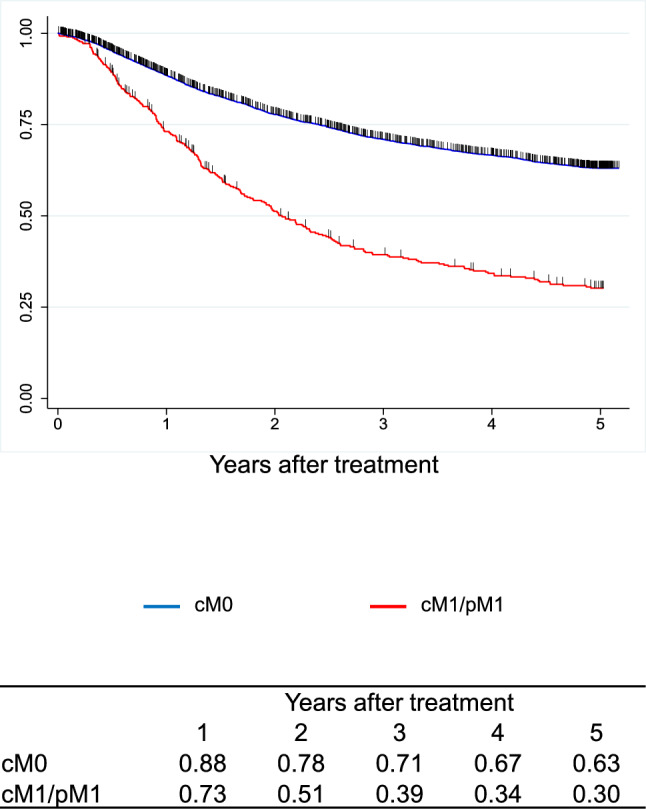
Overall survival of patients who underwent esophagectomy according to distant metastasis (UICC 8.^th^)

Figure 12. Overall survival of patients who underwent esophagectomy according to distant metastasis (UICC 8^th^)

**Fig. 13:**
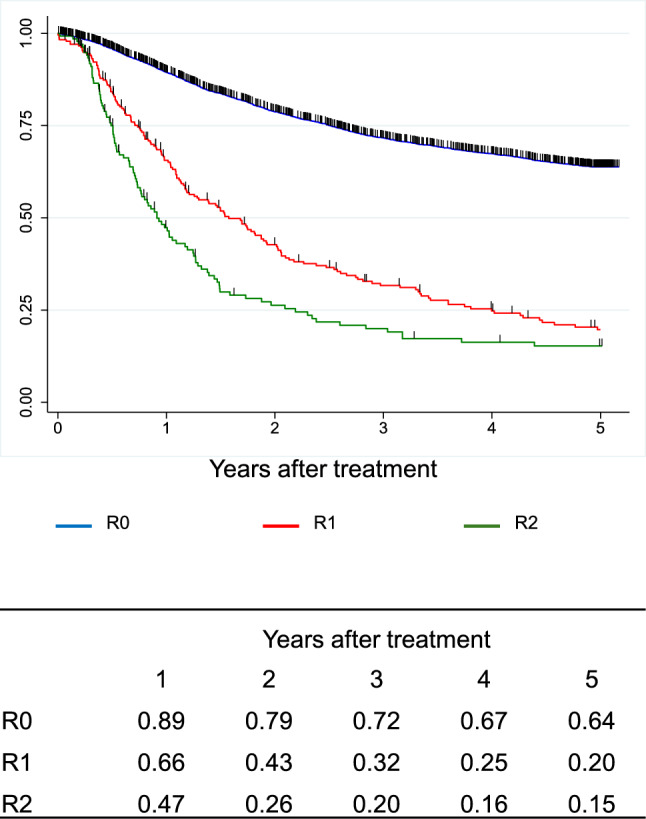
Overall survival of patients who underwent esophagectomy according to residual tumor

Figure 13. Overall survival of patients who underwent esophagectomy according to residual tumor

**Fig. 14:**
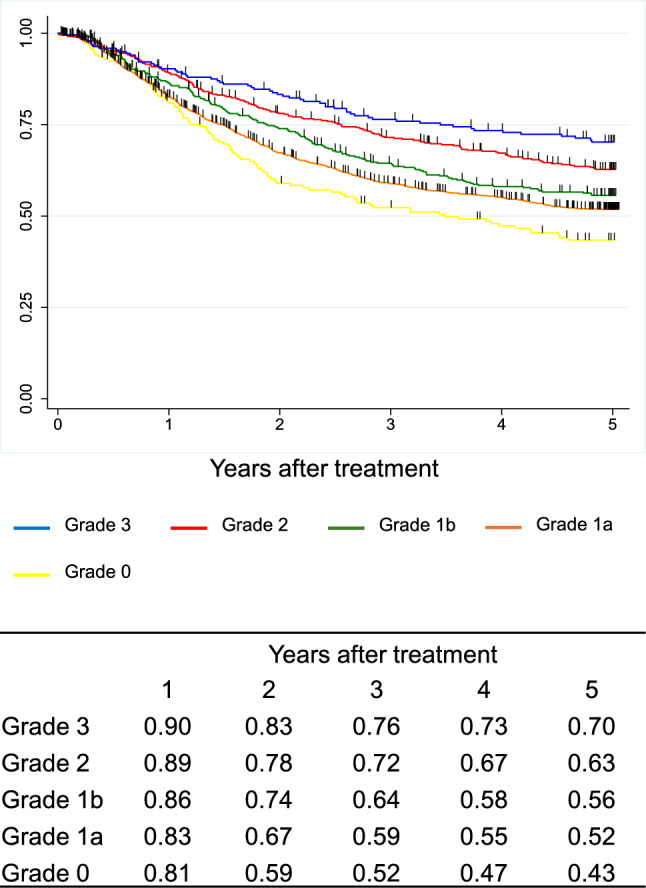
Overall survival of patients who underwent esophagectomy according to histological response to chemotherapy and/or radiotherapy

Figure 14. Overall survival of patients who underwent esophagectomy according to histological response to chemotherapy and/or radiotherapy

I. Clinical features of esophageal cancer patients treated in 2016.
1. Institutions participated in the 2016 registry.

**Table:** 

Institutions
Ageo Central General Hospital
Aichi Cancer Center
Aichi Medical University Hospital
Aizawa Hospital
Akita University Hospital
Aomori Prefectural Central Hospital
Arao Municipal Hospital
Asahikawa Medical University Hospital
Cancer Institute Hospital of JFCR
Chiba Cancer Center
Chiba Central Medical Center
Chiba Medical Center
Chiba University Hospital
Chiba-Nishi General Hospital
Dokkyo Medical University Hospital
Dokkyo Medical University Saitama Medical Center
Edogawa Hospital
Ehime Prefectural Central Hospital
Ehime University Hospital
Eijyu General Hospital
Fuchinobe General Hospital
Fuji City General Hospital
Fujioka General Hospital
Fujisawa City Hospital
Fujita Health University Hospital
Fukaya Red Cross Hospital
Fukui University Hospital
Fukui-ken Saiseikai Hospital
Fukuoka City Hospital
Fukuoka Shin Mizumaki Hospital
Fukuoka University Chikushi Hospital
Fukuoka University Hospital
Fukushima Medical University Hospital
Fukushima Rosai Hospital
Fukuyama City Hospital
Gifu Municipal Hospital
Gifu Prefectural General Center
Gifu University Hospital
Gunma Prefectural Cancer Center
Gunma Saiseikai Maebashi Hospital
Gunma University Hospital
Hachinohe City Hospital
Hakodate City Hospital
Hakodate Goryokaku Hospital
Hakodate National Hospital
Hamamatsu University Hospital
Hasuda Hospital
Heartlife Hospital
Hiraka General Hospital
Hiratsuka City Hospital
Hirosaki University Hospital
Hiroshima City Asa Hospital
Hiroshima City Asa Hospital
Hiroshima Memorial Hospital
Hiroshima Prefectural Hospital
Hiroshima Red Cross Hospital & Atomic-bomb Survivors Hospital
Hiroshima University Hospital
Hitachi General Hospital
Hofu Institute of Gastroenterology
Hokkaido University Hospital
Hospital of the University of Occupational and Environmental Health, Japan
Hyogo Cancer Center
Hyogo Medical University Hospital
Hyogo Prefectural Amagasaki General Medical Center
Ibaraki Prefectural Central Hospital
Iizuka Hospital
Ikeda City Hospital
Imari Arita Kyoritsu Hospital
International University of Health and Welfare Hospital
International University of Health and Welfare Mita Hospital
Iseikai Hospital
Ishikawa Prefectural Central Hospital
Iwata City Hospital
Iwate Medical University Hospital
Iwate Prefectural Central Hospital
Iwate Prefectural Chubu Hospital
Iwate Prefectural Ofunato Hospital
JA Hiroshima General Hospital
JA Kouseiren Enshu Hospital
JA Onomichi General Hospital
Japanese Red Cross Ashikaga Hospital
Japanese Red Cross Fukuoka Hospital
Japanese Red Cross Ishinomaki Hospital
Japanese Red Cross Kitami Hospital
Japanese Red Cross Kyoto Daiichi Hospital
Japanese Red Cross Maebashi Hospital
Japanese Red Cross Medical Center
Japanese Red Cross Musashino Hospital
Japanese Red Cross Nagoya Daini Hospital
Japanese Red Cross Otsu Hospital
Japanese Red Cross Saitama Hospital
Japanese Red Cross Tottori Hospital
Japanese Red Cross Wakayama Medical Center
Japanese Red Cross Yamaguchi Hospital
JCHO Gunma Chuo Hospital
JCHO Kyushu Hospital
JCHO Osaka Hospital
JCHO Saitama Medical Center
JCHO Yokohama Chuo Hospital
Jichi Medical University Hospital
Jichi Medical University Saitama Medical Center
Juntendo University Hospital
Juntendo University Nerima Hospital
Juntendo University Shizuoka Hospital
Junwakai Memorial Hospital
Kagawa Prefectural Central Hospital
Kagawa Rosai Hospital
Kagawa University Hospital
Kagoshima City Hospital
Kagoshima Medical Association Hospital
Kagoshima University Hospital
Kaizuka City Hospital
Kakogawa Central City Hospital
Kanagawa Cancer Center
Kanagawa Prefectural Ashigarakami Hospital
Kanazawa Medical University Hospital
Kanazawa University Hospital
Kansai Denryoku Hospital
Kansai Medical University Hospital
Kansai Medical University Medical Center
Kansai Rosai Hospital
Kanto Central Hospital for Public School Teachers
Kashiwa Kousei General Hospital
Kawasaki Medical School General Medical Center
Kawasaki Medical School Hospital
Kawasaki Municipal Hospital
Kawasaki Municipal Ida Hospital
Kawasaki Saiwai Hospital
Keio University Hospital
Keiyu Hospital
Keiyukai Sapporo Hospital
Kindai University Hospital
Kindai University Nara Hospital
Kinki Central Hospital
Kishiwada City Hospital
Kitaharima Medical Center
Kitakyushu Municipal Medical Center
Kitano Hospital
Kitasato University Hospital
Kitasato University Medical Center
Kobe City Medical Center General Hospital
Kobe City Nishi-Kobe Medical Center
Kobe University Hospital
Kochi Health Science Center
Kochi University Hospital
Kokura Memorial Hospital
Kouseiren Takaoka Hospital
Kumagaya General Hospital
Kumamoto Regional Medical Center
Kumamoto University Hospital
Kurashiki Central Hospital
Kurume University Hospital
Kyorin University Hospital
Kyoto Okamoto Memorial Hospital
Kyoto Saiseikai Hospital
Kyoto University Hospital
Kyoto-Katsura Hospital
Kyushu Central Hospital
Kyushu University Hospital
Matsushita Memorial Hospital
Matsuyama Red Cross Hospital
Mie University Hospital
Minamiosaka Hospital
Minoh City Hospital
Mito Red Cross Hospital
Mitsui Memorial Hospital
Miyazaki University Hospital
Moriguchi Keijinkai Hospital
Murakami General Hospital
Nagahama City Hospital
Nagahama Red Cross hospital
Nagano Municipal Hospital
Nagaoka Chuo General Hospital
Nagasaki Harbor Medical Center
Nagasaki University Hospital
Nagoya City University Hospital
Nagoya City West Medical Center
Nagoya Tokushukai General Hospital
Nagoya University Hospital
Nanpuh Hospital
Nara City Hospital
Nara Medical University Hospital
National Cancer Center Hospital
National Cancer Center Hospital East
National Center for Global Health and Medicine
National Defence Medical College Hospital
Nerima Hikarigaoka Hospital
New Tokyo Hospital
NHO Chiba Medical Center
NHO Iwakuni Clinical Center
NHO Kanmon Medical Center
NHO Kumamoto Medical Center
NHO Kure Medical Center
NHO Kyoto Medical Center
NHO Kyushu Cancer Center
NHO Kyushu Medical Center
NHO Matsumoto Medical Center
NHO Mito Medical Center
NHO Miyakonojo Medical Center
NHO Nagasaki Medical Center
NHO Okayama Medical Center
NHO Osaka Medical Center
NHO Saitama Hospital
NHO Sendai Medical Center
NHO Shikoku Cancer Center
NHO Tokyo Medical Center
NHO Utsunomiya National Hospital
NHO Yokohama Medical Center
Nihon University Hospital
Nihonkai General Hospital
Niigata Cancer Center Hospital
Niigata City General Hospital
Niigata Prefectural Central Hospital
Niigata Prefectural Shibata Hospital
Niigata University Medical & Detal Hospital
Nikko Memorial Hospital
Nippon Medical School Chiba Hokusou Hospital
Nippon Medical School Hospital
Nippon Medical School Tama Nagayama Hospital
Nissan Tamagawa Hospital
Northern Okinawa Medical Center
NTT Medical Center Tokyo
Numazu City Hospital
Obihiro Kousei Hospital
Ofuna Chuo Hospital
Ogaki Municipal Hospital
Ogikubo Hospital
Ohara General Hospital
Ohta Nishinouchi Hospital
Oita Oka Hospital
Oita Prefectural Hospital
Oita Red Cross Hospital
Oita University Hospital
Okayama City Hospital
Okayama Red Cross General Hospital
Okayama Saiseikai General Hospital
Okayama University Hospital
Okitama Public General Hospital
Onomichi Municipal Hospital
Osaka City General Hospital
Osaka General Medical Center
Osaka International Cancer Institute
Osaka Metropolitan University Hospital
Osaka Police Hospital
Osaka Red Cross Hospital
Osaka Rosai Hospital
Osaka University Hospital
Osaki City Hospital
Otemae Hospital
Rinku General Medical Center
Saga Prefectural Hospital Koseikan
Saga University Hospital
Saiseikai Fukuoka General Hospital
Saiseikai Karatsu Hospital
Saiseikai Kawaguchi General Hospital
Saiseikai Niigata Hospital
Saiseikai Noe Hospital
Saiseikai Utsunomiya Hospital
Saiseikai Yamaguchi General Hospital
Saiseikai Yokohama Tobu Hospital
Saitama Cancer Center
Saitama Citizens Medical Center
Saitama City Hospital
Saitama Medical University International Medical Center
Saitama Medical University Saitama Medical Center
Sakai City Medical Center
Saku Central Hospital
Seirei Hamamatsu General Hospital
Sendai City Hospital
Shiga General Hospital
Shiga University of Medical Science Hospital
Shimane Prefectural Central Hospital
Shimane University Hospital
Shin Sapporo Houwakai Hospital
Shin Takeo Hospital
Shinko Hospital
Shinshu University Hospital
Shizuoka Cancer Center
Shizuoka City Shizuoka Hospital
Shizuoka General Hospital
Showa General Hospital
Showa University Hospital
Showa University Koto Toyosu Hospital
Southern Tohoku General Hospital
St. Marianna University School of Medicine Hospital
St. Mary's Hospital
Steel Memorial Yawata Hospital
Suita Municipal Hospital
Tachikawa Hospital
Takatsuki Red Cross Hospital
Teikyo University Chiba Medical Center
Teikyo University Hospital
Teikyo University Hospital Mizonokuchi
Teine Keijinkai Hospital
Tenri Hospital
The Jikei University Daisan Hospital
The Jikei University Hospital
The University of Tokyo Hospital
Tochigi Cancer Center
Toho University Omori Medical Center
Toho University Sakura Medical Center
Tohoku University Hospital
Tokai University Hachioji Hospital
Tokai University Hospital
Tokai University Tokyo Hospital
Tokushima Red Cross Hospital
Tokushima University Hospital
Tokyo Dental College Ichikawa General Hospital
Tokyo Medical and Dental University Hospital
Tokyo Medical University Hachioji Medical Center
Tokyo Medical University Hospital
Tokyo Metropolitan Cancer and Infectious Diseases Center Komagome Hospital
Tokyo Metropolitan Hiroo General Hospital
Tokyo Metropolitan Tama Medical Center
Tokyo Metropolitan Tama-Nanbu Chiiki Hospital
Tokyo Metropolitan Toshima Hospital
Tokyo Women's Medical University Hospital
Tokyo Women's Medical University Yachiyo Medical Center
Tokyo Women's Medical University, Adachi Medical Center
Tonan Hospital
Toranomon Hospital
Tottori Prefectural Central Hospital
Tottori University Hospital
Toyama Prefectural Central Hospital
Toyama University Hospital
Toyohashi Municipal Hospital
Toyonaka Municipal Hospital
Toyota Kosei Hospital
Toyota Memorial Hospital
Tsuchiura Kyodo Hospital
Tsukuba University Hospital
Tsuyama Chuo Hospital
University Hospital, Kyoto Prefectural University of Medicine
University of the Ryukyus Hospital
Wakayama Medical University Hospital
Wakayama Rosai Hospital
Yamagata Prefectural Central Hospital
Yamagata University Hospital
Yamaguchi Rosai Hospital
Yamanashi University Hospital
Yao Municipal Hospital
Yokkaichi Hospital
Yokohama City Municipal Hospital
Yokohama City University Hospital
Yokohama City University Medical Center
Yokosuka Kyosai Hospital
Yuai Memorial Hospital

(Total 347 institutions).

## Supplementary Information

Below is the link to the electronic supplementary material.

Supplementary file1 (DOCX 18 KB)

## References

[1] Japan Esophageal Society. Japanese classification of esophageal cancer, 11^th^ edition: part I. Esophagus. 2017;14:1–36. 28111535 10.1007/s10388-016-0551-7 PMC5222932

[2] Japan Esophageal Society. Japanese classification of esophageal cancer, 11^th^ edition: part II and III. Esophagus. 2017;14:37–65. 28111536 10.1007/s10388-016-0556-2 PMC5222925

[3] Brierley JD, Gospodarowicz MK, Wittekind C, editors. TNM classification of malignant tumors. International Union Against Cancer. 8th ed. Oxford, England: Wiley; 2017.

