# Supplementary material for: Comprehensive registry of esophageal cancer in Japan, 2016
Source: Esophagus. 2025 Jul 6;22(4):475–505. doi: 10.1007/s10388-025-01141-8 (PMC12450814; doi:10.1007/s10388-025-01141-8)
Supplement: Supplementary file 1 — Supplementary file1 (DOCX 18 KB) [file 10388_2025_1141_MOESM1_ESM.docx]

Supplemental Table 1

|  | Synchronous cancer (n=1,260) | | Before esophageal cancer (n=1,277) | | After esophageal cancer (n=609) | |
| --- | --- | --- | --- | --- | --- | --- |
|  | n | % | n | % | n | % |
| Larynx | 68 | 5.4 | 54 | 4.2 | 23 | 3.8 |
| Pharynx | 314 | 24.9 | 150 | 11.8 | 138 | 22.7 |
| Tangue | 25 | 2.0 | 40 | 3.1 | 12 | 2.0 |
| Stomach | 459 | 36.4 | 353 | 27.6 | 97 | 15.9 |
| Colon | 139 | 11.0 | 215 | 16.8 | 61 | 10.0 |
| Liver | 31 | 2.5 | 39 | 3.1 | 21 | 3.5 |
| Biliary tract | 6 | 0.5 | 7 | 0.6 | 5 | 0.8 |
| Pancreas | 20 | 1.6 | 4 | 0.3 | 22 | 3.6 |
| Lung | 93 | 7.4 | 106 | 8.3 | 77 | 12.6 |
| Esophagus | 108 | 8.6 | 119 | 9.3 | 89 | 14.6 |
| Uterine | 3 | 0.2 | 20 | 1.6 | 1 | 0.2 |
| Breast | 3 | 0.2 | 71 | 5.6 | 14 | 2.3 |
| Prostate | 21 | 1.7 | 128 | 10.0 | 35 | 5.8 |
| Bladder | 12 | 1.0 | 62 | 4.9 | 25 | 4.1 |
| Hematopoietic organs | 5 | 0.4 | 29 | 2.3 | 15 | 2.5 |
| Skin | 1 | 0.1 | 9 | 0.7 | 4 | 0.7 |
| Brain | 0 | 0.0 | 1 | 0.1 | 3 | 0.5 |
| Thyroid | 16 | 1.3 | 8 | 0.6 | 4 | 0.7 |
| Bone | 1 | 0.1 | 0 | 0.0 | 1 | 0.2 |
| Kidnery | 6 | 0.5 | 35 | 2.7 | 10 | 1.6 |
| Others | 56 | 4.4 | 70 | 5.5 | 43 | 7.1 |
| Unknown | 3 | 0.2 | 1 | 0.1 | 4 | 0.7 |
|  |  |  |  |  |  |  |
|  |  |  |  |  |  |  |
